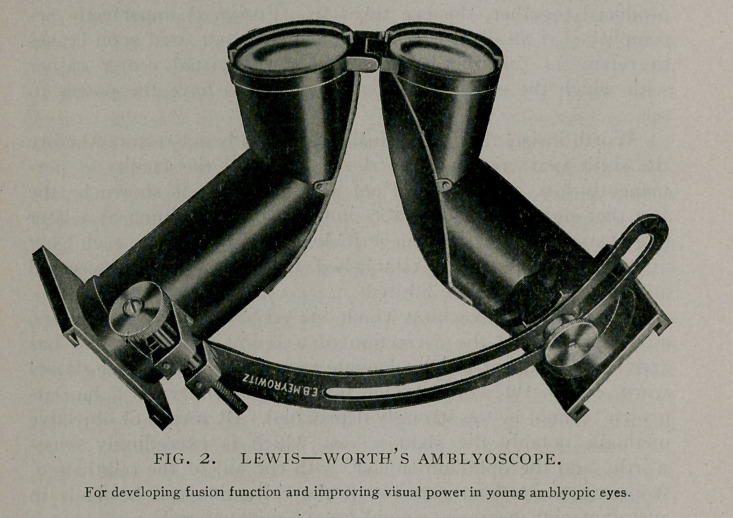# Squint and Its Modern Treatment1Read before the Batavia Medical Club, July 1, 1903.

**Published:** 1903-11

**Authors:** F. Park Lewis

**Affiliations:** Buffalo, N.Y.


### Squint and its Modern Treatment.[1]

By F. PARK LEWIS, M. D., Buffalo, N. Y.

THERE is, perhaps, in ophthalmology no more simple, and at the same time no more difficult subject than that of strabismus; simple, because its correction is most easy, requiring merely the division of a superficial tendon, an operation any surgeon can perform; difficult, because a simple tenotomy, if not considered in connection with the conditions that have caused the squint, will be followed untimately and almost inevitably by a state worse than that which it was designed to correct.

When we remember that a permanently inturned eye becomes at last blind, we have a reason additional to the obstrusive deformity for applying the wisest measures for its relief, at a period when help may yet be afforded. To understand the nature of squint we must know the process by which binocular vision is obtained, and although it may seem elementary it will make our subject more clear if we cansider for a moment how we see.

Of course, paradoxical as it may seem, we do not see with our eyes. They are the media merely through which the impressions made by light waves, are carried to the visual center in the brain. Our eyes are two cameras by which the object seen is photographed by a mechanism and a process almost exactly analogous to that employed in the artist's studio. Indeed, the impression has been "fixed" on the retina as it is on the chemically prepared plate and plainly seen after the tissue has been removed from the eye. For every nerve ending in the retina is a corresponding terminal neuron in the cuneus, and as there is a macula, or central point of keenest vision in the eye, so there must be a corresponding *brain macula* or receptive point of greatest acuteness in the cerebral cortex. The impression made by the picture upon each retina is carried to both sides of the cerebral hemispheres, but in unlike degree as the temporal field in each eye is the greater. There is consequently an exact over-lapping

1. Read before the Batavia Medical Club, July 1, 1903.

of the impression of the image carried from each eye to each side of the brain.

It will be seen then that in order that there be perfect correspondence between the impressions on each visual cortex, there must be perfect harmony between the two mechanisms by which these impressions are focused. The human eye, almost immediately adjusts itself to any focal range within its possibilities. This is not accomplished by means of a screw as in a camera, but by, at least, eighty muscular processes constituting the ciliary body,

FIG. I. LEWIS—DIAGRAMATIC SKETCH OF THE CORRESPONDING NUCLEAR AND CILIARY NEURONS.

R. C.—Right ciliary region. L. C.—Left ciliary region. 1, 2, 3, 4, 5, etc.—Neurons governing corresponding accommodative ciliary fibers. R. A. C.—Right center for accommodation. L. A. C.—Left center for accommodation. A. F.—Association fibers connecting corresponding nuclear neurons.

and these are each again subdivided into individual fibers, so that there are certainly as many single living chords controlling the focus of the lens, as there are degrees in a circle.

The center for accommodation has been located as a part of the group of cells, constituting the nucleus of the third nerve. As it has been shown that these ciliary elements act synchronously, there must be nuclear origins for each fiber, and consequently the center for accommodation is not a point but a multitude of neurons. (See Fig. 1).

The various parts, therefore, of the mechanism of vision are so interrelated that to work harmoniously they must be anatomically symmetrical and normal. If one eye is too short in its long axis, as occurs in hypermetropia, or if it is too long, as in myopia, or if the axes are incommensurate, as in astigmia, or if the eyeballs themselves are unsymmetrical as in anisometropia, or as it is essential that the visual axes shall be both directed toward the object observed, if the muscles are in a state of imbalance as in heterophoria, then the effect exerted upon the delicate muscle fibers, in an endeavor to correct by adjustment that which is organically wrong, may disturb the harmony of action and affect both the visual centers as well as centers associated with them.

Now the infant does not possess at birth the function of binocular fixation. The eyes roll aimlessly for the first six weeks of life and only gradually acquire the power of working coördinately. The complete power of fusing images has usually been acquired by the sixth year according to Worth[1], whose recent book is the most valuable contribution to this subject for many years, and to whom I am indebted for many of the important conclusions which I shall endeavor to outline in this paper.

Among all of the complicated and accurately balanced coordinate actions, there are none in which a nicer adjustment is required than in that of seeing. It is first necessary that the cortical center governing the muscles of the neck be energised in order that the head be lifted to the right angle and turned in the proper direction toward which the eyes are to be directed. If the object is within a short range the center for convergence must cause the internal recti to pull the eyes in exactly the position that will make the visual lines cross in the plane of the object to be seen. Meanwhile the externi are holding the balance as guy ropes in order that the convergence be not excessive. In any slight rotation the obliques are working as pairs or antagonists. Coincidently the accommodative centers, which as has been shown, have a hundred or more inter relationships, cause the ciliary muscle to give the exact degree of curvature to the lens to bring the focus upon the desired point.

That these involved actions may be performed at the same time or in rapid succession as may be desired, each of the centers must be directly connected by means of association fibers with each of the others and the combined activities produce the function of fusion. That is to say, a stereoscopic effect is produced by the impressions of both eyes being superimposed in the cortex of each cuneus so exactly that as undoubtedly the cortical neurons are also connected, the sensation is excited in the brain of a single image and we have binocular vision with perfect fusion of the images.

1. Squint—Its Causes, Pathology and Treatment. By Claud Worth, F. R. C. S., 1903.

If, however, as frequently happens, the mechanism by which the two foci are produced is not alike, the images are not then superimposed nor produced with equal clearness. One camera,—one eye,—may be longer or shorter in its focus than the other, or the radii of corneal curvature may be greater or less in one meridian than in the corresponding meridian of the fellow eye. The images will then be unlike on the retinæ and the impressions uneven on the cuneus cortex. In that hemisphere, therefore, corresponding to the defective eye the impression is dull. Greater effort is made by the centers governing accommodation to get a clear impression and, as accommodation and convergence are produced together, the eye turns in. Fusion is imperfectly accomplished if at all. As a muscle which is not used soon ceases to retain its function, so an eye, or the visual center rather with which the eye communicates, ceases to have the power to see.

Worth insists that if this fusion function is not restored before the sixth year, vision never is regained and the faculty is permanently lost. That this is not absolutely true is shown by the fact that sight does measurably improve under training at a later age than this; but it is, nevertheless, true that with each year after the squint becomes established, the difficulties are increased and the possibilities are limited.

The pernicious teaching which one yet occasionally encounters, advising delay in the correction of a squint until the child has arrived at an age at which adequate tests may be made and glasses worn, or the yet worse advice that the deformity "will be outgrown" cannot be too strongly deprecated. By means of objective methods, notably the shadow test which is exceedingly trustworthy and the ophthalmometer, with the aid of the ophthalmoscope, an exact determination of the refraction can be made in an infant and measures should be at once undertaken to correct the existing defect on which the squint depends.

The first requirement may be to use a cycloplegic in the better eye. This measurably throws that eye out of commission and compels the use of the defective and squinting one. The refraction should be at once corrected; glasses may be worn by a very young child and the relief is great and immediate. To train the fusion faculty Mr. Worth has devised an immensely practical and useful instrument which he calls the amblyoscope[1]. It consists of two-hinged tubes so arranged with mirrors that binocular vision may be obtained when the visual angle has a convergence of 60 degrees or a divergence of 30 degrees. It is therefore, possible if poten-

1. See Fig. 2, p. 252.

tial fusion exists to bring the visual axis together and after correcting unequal refraction to compel the defective eye to make the effort to see.

It is astonishing how rapidly an amblyopic eye may be trained to a point of normal visual acuity by this method, and a defect that must otherwise soon become permanent and established be overcome.

The conclusions therefore, are:

1. That no period is too early for the recognition and attempted correction of a threatened or imminent squint.

FIG. 2. LEWIS—WORTH'S AMBLYOSCOPE.

For developing fusion function and improving visual power in young amblyopic eyes.

2. That when recognised, refractive differences should be adjusted at the earliest period at which glasses may be worn.

3. That fusion training should be at once employed under the direction of the surgeon, in order that incipient or established amblyopia may be corrected before the defect becomes fixed and incurable.

4. That surgical intervention should be undertaken as soon as its necessity becomes established, and should consist of tenotomy or advancement or both, at such a time as will permit perfect fusion, if correction with glasses and fusion training by the amblyoscope will not suffice to correct the existing deformity.

454 FRANKLIN STREET.

## Figures and Tables

**Fig. I. f1:**
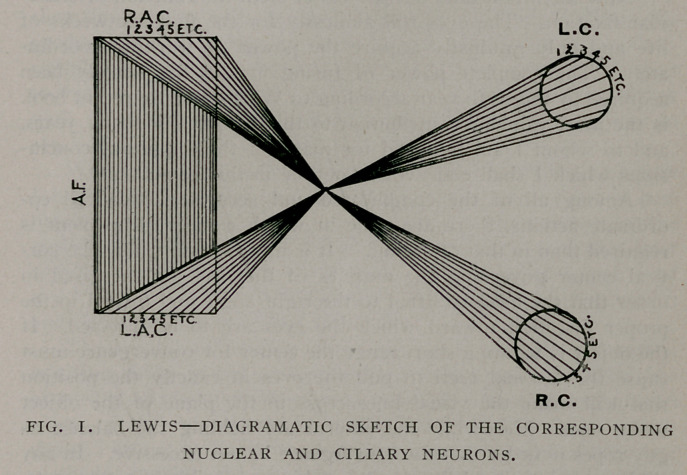


**Fig. 2. f2:**